# Development of an interdisciplinary early rheumatoid arthritis care pathway

**DOI:** 10.1186/s41927-022-00267-x

**Published:** 2022-06-25

**Authors:** Xenia Gukova, Glen S. Hazlewood, Hector Arbillaga, Paul MacMullan, Gabrielle L. Zimmermann, Cheryl Barnabe, May Y. Choi, Megan R. W. Barber, Alexandra Charlton, Becky Job, Kelly Osinski, Nicole M. S. Hartfeld, Marlene W. Knott, Paris Pirani, Claire E. H. Barber

**Affiliations:** 1 Department of Medicine, Cumming School of Medicine, Faculty of Medicine, University of Calgary, 3330 Hospital Drive, NW, Calgary, AB T2N 4N1 Canada; 2 Department of Community Health Sciences, Cumming School of Medicine, University of Calgary, Calgary, AB Canada; 3 Arthritis Research Canada, Vancouver, Canada; 4 Alberta Health Services, Calgary, Canada; 5 Learning Health System, Alberta SPOR SUPPORT Unit, Alberta, Canada

**Keywords:** Arthritis, Rheumatoid, Quality of health care, Quality improvement

## Abstract

**Background:**

To develop an interdisciplinary care pathway for early rheumatoid arthritis (RA) including referral triage, diagnosis, and management.

**Methods:**

Our process was a four-phase approach. In Phase 1, an anonymous survey was electronically distributed to division rheumatologists. This provided data to a small interprofessional working group of rheumatology team members who drafted an initial care pathway informed by evidence-based practice in Phase 2. In Phase 3, an education day was held with approximately 40 physicians (rheumatologists and rheumatology residents), members of our interprofessional team, and two clinic managers to review the proposed care elements through presentations and small group discussions. The care pathway was revised for content and implementation considerations based on feedback received. Implementation of the care pathway and development of strategies for evaluation is ongoing across multiple practice sites (Phase 4).

**Results:**

Our care pathway promotes an approach to patient-centered early RA care using an interdisciplinary approach. Care pathway elements include triage processes, critical diagnostics, pre-treatment screening and vaccinations, and uptake of suggested RA pharmacologic treatment using shared decision-making strategies. Pathway implementation has been facilitated by nursing protocols and evaluation includes continuous monitoring of key indicators.

**Conclusion:**

The ‘Calgary Early RA Care Pathway’ emphasizes a patient-centered and interdisciplinary approach to early RA identification and treatment. Implementation and evaluation of this care pathway is ongoing to support, highest quality care for patients.

**Supplementary Information:**

The online version contains supplementary material available at 10.1186/s41927-022-00267-x.

## Background

Several guidelines have been created for the pharmacologic management of rheumatoid arthritis (RA) [1–3]. While important to informing best practices, guideline implementation often falls short [4–6]. Furthermore, there remain many unmet needs for individuals with RA particularly in management of pain, fatigue, and physical function [7]. RA is also associated with a high psychosocial burden, which can add complexity to care and negatively impact outcomes [8]. There is an increasing call to incorporate more holistic management strategies to RA to better address these complex patient needs [9], further emphasizing the central role of an interdisciplinary treatment plan.

A care pathway is commonly defined as “a complex intervention for the mutual decision-making and organization of care processes for a well-defined group of patients during a well-defined period” [10]. A care pathway facilitates communication and care coordination among multidisciplinary team members, patients, and families. Care pathways can also be used to help define and coordinate care team member roles, care processes, aid in documenting, monitoring and evaluating outcomes[10]. They can also help to identify appropriate resources needed for optimal care delivery [10]. Care pathways should standardize care and facilitate shared decision-making when appropriate [11]. Four criteria have been proposed for the operational definition of a care pathway including the following pathway characteristics [12]: i) “structured multidisciplinary plan of care”; ii) translation of guidelines or evidence for local application; iii) steps of care or treatment in a plan/pathway/algorithm etc.; and iv) an overall aim to standardize care for a specific population.

Care pathways can improve patient outcomes and reduce unwarranted variation in care. There are many examples of successful care pathway implementation which have led to improvements in care processes and patient outcomes including: in gout management [13], cardiology [14, 15], nephrology [16], and fracture management [17], to name a few. In Alberta, Canada, physicians have access to care pathways for common conditions and rapid telephone advice for additional support through Specialist Link [18, 19]. The Primary Care Gout Pathway [20], is one such pathway that has been developed to standardize treatment strategies and achieve better patient outcomes in gout. These pathways are integral to supporting physicians in the management of diseases with the aim of improving diagnosis, early treatment and optimizing management and patient outcomes and reducing the need for costly in-person specialist consultations. Unfortunately, a standardized approach to holistic early RA management including screening for complications of RA is lacking. Our aim was to develop an interdisciplinary care pathway for early RA to promote best practices for referral triage, diagnosis, and management.

## Methods

### Overall approach

When designing a complex intervention such as a care pathway, one or more implementation frameworks are used to guide the process and the application of implementation strategies is often dynamic and iterative in nature. As a foundation for this process, we used the Knowledge-to-Action (K2A) framework [21], also known as a “Process Model”[22] to help specify stages of the translation of guidelines and best practices into a care pathway. The K2A framework has two main parts: knowledge creation parts and the action cycle which comprises seven phases [21]. Within the K2A framework, understanding the domains and individual determinants of barriers and facilitators to implementation is also important, and “Determinant Frameworks””[22] can be further used to identify and understand important determinants. The Consolidated Framework for Implementation Research (CFRI)[23] is a determinant framework that served as a practical guide for identifying facilitators and barriers for implementing the elements of the care pathway and its use is ongoing during our implementation efforts. Lastly, the strategies to address barriers to implementation were identified through linkage to Expert Recommendations for Implementing Change (ERIC)[24]. Below in the description of the methods we have highlighted the linkage to the K2A framework employed at each phase.

**Fig. 1:**
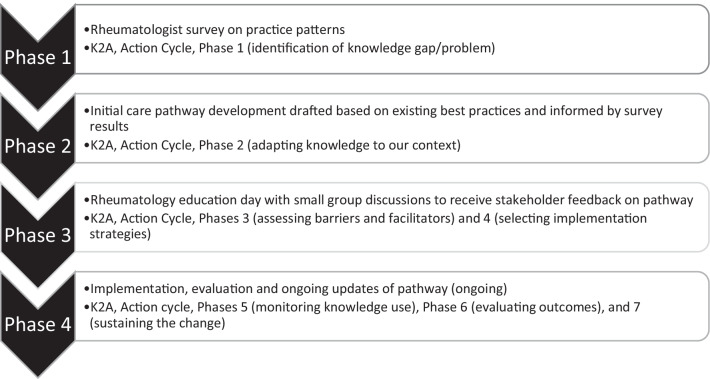
Phases of early rheumatoid arthritis care pathway development and alignment with knowledge to action cycle (K2A) phases

The care pathway was developed in four main phases, visualized in Fig. 1. In Phase 1, a practice patterns survey was distributed to better understand practice variability in early RA management among our rheumatologists and to identify gaps between knowledge and practice (K2A Phase 1 [21]). Phase 2, an initial care pathway and accompanying clinical guidance document was drafted to adapt best practices and guidelines to our local context (K2A Phase 2 [21]). The guidance included best practices according to current guidelines [1–3, 25], and a consensus approach in areas where evidence was lacking (e.g., specific glucocorticoid dosing for flares, dosing of folic acid, etc.), which was informed by the results of the practice patterns survey. In Phase 3, an education day was held with small group discussions to review the draft pathway and advise on content and implementation enhancements to identify barriers and facilitators to practice change (K2A Phase 3, [21]) and to develop implementation strategies (K2A Phase 2 and 4 [21]); in Phase 4 (ongoing), the pathway is being implemented and evaluated across multiple clinical sites (K2A Phase 5–7 [21]).

### Phase 1: current practice patterns

The Division of Rheumatology in Calgary, Alberta Canada consists of 21 rheumatologists at university-based clinics and 15 rheumatologists at community-based clinics serving adults with inflammatory rheumatic diseases. The catchment area for the Division includes the southern half of Alberta and extends into southeastern British Columbia and southwestern Saskatchewan, comprising a service population of approximately 2.3 million individuals [26]. The Division members also provide inpatient consultation at four hospitals in the Calgary a Zone of Alberta Health Services. Our division at present does not include a patient advisory board.

An anonymous practice patterns survey was electronically distributed to physician members of our division (survey available upon request, results shown in Additional File 1). The questionnaire was developed by two rheumatologists (CEHB and GH) and a research assistant (NS), and pilot tested prior to administration. It included 57 questions regarding physician early RA practice patterns including the typical choice of initial disease-modifying therapy; route, dose, and titration practices for disease modifying anti-rheumatic drugs (DMARDs); dose and frequency of folic acid; choice of route and dose of corticosteroids; initial imaging; baseline labs and frequency of monitoring; vaccinations; and medication counselling. A combination of 9-point Likert scales assessing the likelihood of practices such as prescribing various DMARD combinations, yes/no answers, and free-text comments were used for responses. Ethics approval was obtained for the study from the University of Calgary Research Ethics Board, and all participants provided informed consent (REB-19–1080).

### Phase 2: initial draft of the early RA care pathway

Using data acquired from the practice survey and best practices from current guidelines [1–3, 25], an initial care pathway was drafted by a small working group including three rheumatologists at an urban academic practice, one rheumatology resident, one community rheumatologist, one nurse manager, two nurses, one physiotherapist, one pharmacist and one social worker. These members were engaged to identify existing clinic protocols (e.g., triage protocols) and resources (e.g., educational programs) and these were incorporated into the working draft. While we did not have patient representation in our small working group, we leveraged our prior work incorporating patient perspectives and representation on various elements included in the pathway including: central triage [27, 28], patient decision aids and shared decision making [11], defining high quality of care in RA [29], and measuring quality of care in RA [30, 31].

### Phase 3: group discussions

An education day was held with approximately 40 physicians (including rheumatologists and rheumatology residents) and members of our interprofessional team (nursing, social work, physiotherapy, and pharmacy providers) and two clinic managers. Following a presentation, the attendees were divided into break-out groups of 10 to12 individuals, facilitated by trained support staff (three implementation support experts). A semi-structured guide of questions on five main care pathway topics along with probes was developed. The questions were directed at understanding (1) whether the care pathway reflected current practice and if there were any gaps identified; (2) how to optimally implement elements of the care pathway; and (3) if there were barriers/facilitators identified. While the groups were not recorded, detailed notes were taken with support from our implementation experts. Following break-out room discussions, a large group session was held to reflect upon the comments as a group. Barriers and facilitators to care pathway implementation were identified through mapping to CFRI [23] domains (see Additional File 1) and implementation solutions were identified through linkage to ERIC recommendations [24]. The care pathway was revised based on feedback and distributed for implementation.

### Phase 4: implementation and evaluation

The care pathway is summarized in a 20-page document outlining our team approach to early RA care. An accompanying 14-page document was also developed to support nurses in answering telephone calls from patients on common issues (both documents available upon request). The documents were distributed to team members and will be updated regularly as required. Implementation of the pathway is ongoing and has begun at the largest university-based site. To facilitate implementation of the pathway, nurse case manager roles were developed to support patient care. Team meetings are ongoing to evaluate care delivery and identify areas for further optimization. Our site is undergoing a transition to a provincial electronic medical record (EMR) and once this is complete evaluation of key quality metrics will be developed for dashboard display of quality metrics.

## Results

### Initial pathway development (Phase 1–3)

During Phase 1, there were 16 respondents to our practice pattern survey (40% response rate). The main results of the survey are shown in Additional file 1: Tables S1–S7. While the results demonstrated strong similarities in the practice patterns of rheumatologists in the management of early RA, there was some variability in practice on almost all questions asked highlighting a need for a standardized approach to early care and treatment. The results of this work helped to inform the need for a care pathway and important areas of variability to address. It also identified common resources used by physicians for medication counselling and healthcare team members participating in treatment counselling and management. Following initial care pathway development (Phase 2), feedback on the care pathway was obtained during our education day. Barriers and facilitators to implementation of key pathway elements were mapped (Additional file 1: Table S8), which helped inform our initial implementation strategy.

### Calgary early RA care pathway

The following sections describe the content elements in the Calgary Early RA Care Pathway based on the information gathered from Phases 1–3.

### Triage process

The current central triage process for early RA was implemented in 2006 [32]. Ambulatory care referrals are received in a central office and reviewed for reason for referral and accompanying investigations by trained nurses with rheumatologist oversight. General referral requirements include: reason for referral, medication list, history, physical exam, and attempted treatments. Referral requirements specific to inflammatory arthritis include a complete blood count (CBC), C-reactive protein (CRP), liver enzymes, kidney function, a rheumatoid factor (RF) and anti-cyclic citrullinated peptide antibody (anti-CCP) completed within the prior three months. Information required for the referral, including a referral for suspected inflammatory arthritis, is available on the Alberta Referral Directory, a website housed by Alberta Health Services [33]. Criteria considered when assessing the urgency of a referral for early RA are shown in Table 1. These include: (1) laboratory findings including presence of an elevated anti-CCP, RF, and/or elevated CRP; (2) physical examination findings by the referring physician including symmetrical joint pain or joint swelling on exam, and presence of psoriasis (in case of psoriatic arthritis) (3) important items on history including morning stiffness lasting more than 30 min, symptoms lasting more than 6 weeks, and positive family history of RA.

**Table 1:** Criteria for referral evaluation

General features	Urgent rapid assessment (< 4 weeks)	Urgent assessment (1–3 months)
Elevated anti-CCP	3 or more features	< 3 features
Elevated RF		or
Elevated CRP		Seronegative with joint swelling on exam
Description of symmetrical joint pain and joint swelling on exam		or
AM stiffness > 30 min		Description suggestive of psoriatic arthritis (e.g., history of psoriasis)
Symptoms > 6 weeks		
Positive family history		

Anti cyclic citrullinated peptide antibody (anti-CCP), rheumatoid factor (RF), C-reactive protein (CRP)

The clinical judgement of triage nursing staff and physicians is used to assess the urgency of these referrals. As a guide, patients with three or more of these criteria are triaged as possible early RA and waitlisted as “urgent priority” (< 4 weeks). Patients with fewer than three criteria are waitlisted as “urgent” (1–3 months). Individuals that are seronegative but have joint swelling including those with psoriasis (e.g., possible psoriatic arthritis) are waitlisted as urgent (1–3 months). If serology or physical exam findings are missing such that the referral cannot be appropriately triaged, the referral is redirected back to the referring physician and a request is made to clarify the missing information needed to complete and resubmit the referral.

Additional rheumatology access occurs through Specialist Link and on-call services. Specialist link provides physician to physician telephone advice to support outpatient specialty advice (including rheumatology) and expedite referrals in the Calgary zone [18, 19]. Rheumatologists are also available for inpatient consultation at four major Calgary area hospitals.

### Early RA workup

Referred patients meeting criteria for possible early RA are assessed by the next available rheumatologist. Once the patient is seen in clinic, our early RA workup includes baseline laboratory tests and imaging if not already completed by the primary care physician. The tests and rationale for their inclusion are shown in Table 2. Routine testing of Erythrocyte Sedimentation Rate (ESR) has been discouraged by Alberta Health Services provincial lab directors since 2013 in favour of using the CRP, given the higher specificity and sensitivity and better analytic performance of this test [34, 35]. Rheumatology providers still have access to ESR testing in our province if they feel it would be useful to monitor disease activity.

**Table 2:** Early rheumatoid arthritis baseline work-up

Laboratory investigations	Rationale
CBC	Evaluate for any baseline abnormalities including cytopenia (e.g., anemia of chronic disease, neutropenia)
Serology: RF, anti-CCP	Assist with the diagnosis and prognosis of early RA
Inflammatory markers: CRP, ESR *	Assist with ascertaining diagnosis and disease activity
Additional serology: ANA, ENA	May be warranted in certain clinical scenarios to ascertain overlap syndromes or alternative diagnoses
Renal function: creatinine/GFR, urinalysis	Identify pre-existing renal disease which could complicate therapy
Liver function: albumin, ALP, ALT	Identify pre-existing liver disease which could complicate therapy
Hepatitis screening: Hepatitis B Sag, Hepatitis B Sab, Hepatitis B Core, HCV	Identify pre-existing hepatitis which could complicate therapy
HIV	Depends on risk factors
QuantiFERON	Screen for tuberculosis
Imaging**	
X-ray of hands/wrists	Identify damage from RA and evaluate any other findings indicative of an alternative diagnosis (e.g., psoriatic arthritis, gout, osteoarthritis)
X-ray of ankles/feet	Identify damage from RA and evaluate any other findings indicative of an alternative diagnosis (e.g., psoriatic arthritis, gout, osteoarthritis)
Chest X-ray	Screen for tuberculosis and other pre-existing lung pathology (e.g., ILD)

Complete Blood Count (CBC), Rheumatoid Factor (RF), Anti Cyclic Citrullinated Peptide Antibody (anti-CCP), C-Reactive Protein (CRP). Erythrocyte Sedimentation Rate (ESR), Anti-Neutrophil Antibody (ANA), Extractable Nuclear Antigen (ENA), Glomerular Filtration Rate (GFR), Alkaline Phosphatase (ALP), Alanine Aminotransferase (ALT), Hepatitis C virus (HCV), Surface Antigen (Sag), Surface Antibody (Sab), Interstitial Lung Disease (ILD), Tuberculosis (TB)

^*^Tested at the discretion of the attending rheumatologist

^**^Additional imaging on a case-by-case basis

### Screening baseline laboratory investigations

In addition to the above, early RA investigations include screening for chronic hepatitis (hepatitis B surface antigen, surface antibody, core antibody; hepatitis C) in all individuals and HIV in individuals with HIV risk factors [36] (Table 2).

Screening for tuberculosis (TB) may also be included in early RA work-up. This includes QuantiFERON test (or less commonly a skin test), and a chest radiograph. In particular, TB screening is recommended in individuals who may require high dose glucocorticoids (ideally prior to initiation), as this is an important risk factor for reactivation [37]. Screening should also occur routinely at baseline in individuals with additional risk factors for TB (e.g. patients from TB-endemic areas, injection drug users, patients with no fixed address, healthcare workers, travelers etc. [37]). In individuals with prior treated TB or treated latent TB if records were not available, TB specialist consultation is requested. TB screening should be completed at the initial visit in all individuals with features of poor prognosis who are at high risk of rapid progression to a biologic agent (e.g., RF/anti-CCP positivity, functional limitation, high number of swollen and tender joints, high inflammatory markers, early erosions, and extraarticular features)[25].These recommendations, in addition to other guidelines on pre-biologic screening for TB, may help avoid delays in biologic starts for individuals.

### Vaccinations

Early review of vaccination status is recommended in the pathway and all patients are provided written information on available and recommended routine vaccinations. Vaccine recommendations are discussed with patients, and they are provided written information and guidance to further review recommendations with their primary care provider, a public health nurse and/or a pharmacist. Recommended vaccinations are listed in Table 3 and these recommendations are in line with European League Against Rheumatism (EULAR) vaccination guidelines [38, 39]. Although not available for discussion at the initial draft of the care pathway, COVID19 mRNA vaccination is also recommended [40], and information from the Canadian Rheumatology Association (CRA) [41] has subsequently been incorporated into patient discussions and information packages in the clinic.

**Table 3:** Recommended vaccinations [40, 60, 61]

Vaccine type	Candidates for vaccine	Notes
Influenza	Annually for everyone	Flumist should not be given to patients on immune modifying medication
	Family members and close contacts should also receive the vaccine	
	Consider High Dose Vaccine for those ≥ 65 years	
Pneumo-13 (Prevnar)	Any patient on DMARDs, biologics or immunosuppressants	Should be given 8 weeks prior to Pneumovax OR at least 12 months following Pneumovax
Pneumo-23 (Pneumovax)	Everyone age ≥ 65	If both doses were given prior to age 60, consider a 3rd dose after age 65
	All patients on DMARDs, biologics or immunosuppressants, regardless of age	
	Immunocompromised patients should receive a booster in 5–10 years	
Varicella Zoster (Shingrix)	Everyone age ≥ 50, especially those who are going to be receiving a biologic medication or JAK inhibitor	Those who have previously had the live vaccine or those who have had shingles previously can receive this vaccine after at least 1 year has passed
Hepatitis A and B	For those at high risk (e.g., travel to or residence in endemic countries for hepatitis A and/or B); increased risk of exposure or proven exposure to hepatitis A and/or B (e.g., because of medical profession, infected family member or contacts)	
COVID-19 vaccination	Everyone	Guidance is evolving in this area. Current CRA guidance [40] suggests vaccination with any of the currently available COVID-19 vaccines. For those on Rituximab immunization should occur > 4–5 months after the last dose and at least 4 weeks prior to the subsequent dose. Current CRA guidelines do not recommend holding DMARDs for vaccination. A third dose of COVID-19 mRNA vaccination are currently suggested [62] for individuals immunosuppressants which could impact response (e.g., rituximab, mycophenolate mofetil, JAK inhibitors, abatacept, anti-TNF agents, antimetabolites etc.)

Anti-Tumor necrosis factor alpha (anti-TNF); Canadian rheumatology association (CRA); Janus kinase inhibitor (JAK inhibitor)

### Early RA treatment

**Fig. 2:**
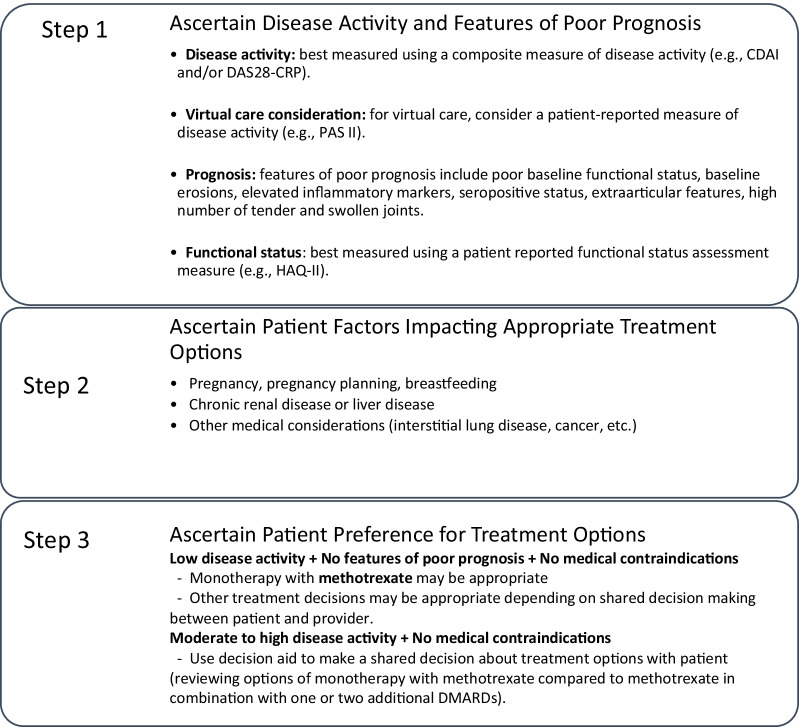
Choice of DMARDs for treatment of early rheumatoid arthritis. Clinical Disease Activity Index (CDAI), Disease Activity Score-28 C-Reactive Protein (DAS28-CRP), Disease Modifying Anti-Rheumatic Drugs (DMARDs), Health Assessment Questionnaire-II (HAQ-II), Patient Activity Scale-II (PAS-II)

Early and targeted treatment strategies in RA have become a paradigm for care [3, 25, 42], and are associated with improved outcomes, including lower disease activity, improved function, and less radiographic damage [43–47]. These treatment options are summarized in Fig. 2.

Consistent with guidelines, the care pathway includes methotrexate either orally or subcutaneously as the first DMARD, either alone or in combination with other DMARDs (e.g., sulfasalazine and hydroxychloroquine), made as a shared decision between the patient and rheumatologist. In most clinical situations, the starting dose of oral methotrexate is 15 mg PO weekly to assess tolerability, then increased to 20 mg weekly. The starting dose of subcutaneous methotrexate is 20 mg weekly. Lower starting doses of methotrexate may be appropriate in some populations of patients at the discretion of the rheumatologist. Folic acid is prescribed concomitantly with methotrexate and is recommended in a dose of 5 mg daily. Sulfasalazine is started at 500 mg twice daily, with a gradual titration to 2 g total daily dose within 4 weeks. Hydroxychloroquine is dosed at 5 mg/kg (actual body weight) daily (to a maximum of 400 mg/d) as per guidelines from the American Academy of Ophthalmology to decrease ocular toxicity [48]. Leflunomide is started at 20 mg/d in most individuals (lower dosing may be appropriate if there is a higher concern for the potential of toxicity at the discretion of the rheumatologist).

**Fig. 3:**
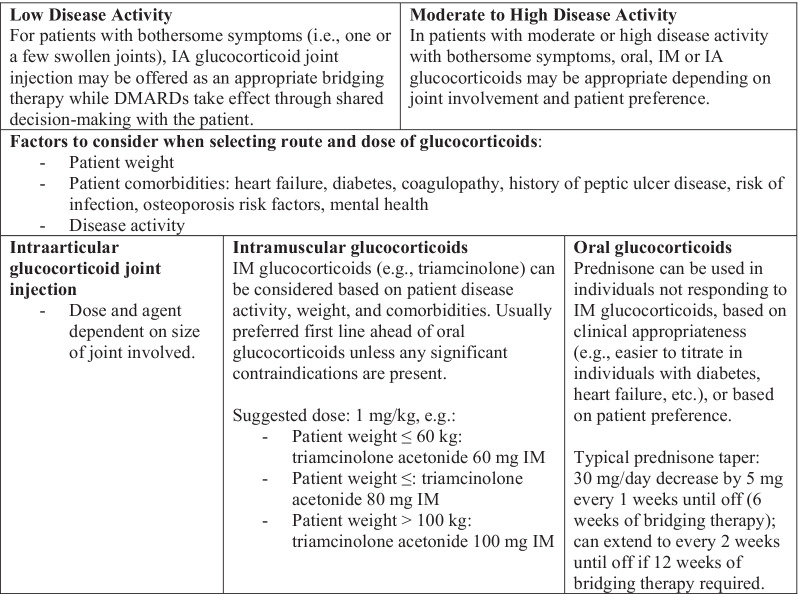
Choice of corticosteroids. Disease modifying anti-rheumatic drug (DMARD); Intraarticular (IA); Intramuscular (IM)

Glucocorticoids are commonly used in early RA to treat disease manifestations acutely while slower-acting DMARDs take effect. Specific guidance on dose/use of steroids (dependent on disease activity, patient preference, and any comorbid conditions) is summarized in Fig. 3.

Regular monitoring of functional status and disease activity (typically at each visit) is included in the pathway to help monitor disease and target treatment. Currently, most rheumatologists in our division use the CLINHAQ (Clinical Health Assessment Questionnaire), which consists of a HAQ-DI (Health Assessment Questionnaire Disability Index) and includes some additional questions on fatigue, stiffness, and gastrointestinal distress. However, recently the American College of Rheumatology (ACR) released guidance about recommended functional status measures and, because of its psychometric properties, the HAQ-DI is no longer recommended [49]. Measures recommended include the PROMIS physical function 10 item short form (PROMIS PF10a), the HAQ- II, and the Multidimensional HAQ [49]. This guidance has been recently updated for use in virtual care [50]. The HAQ-DI and DAS28 are currently required for advanced therapy coverage by provincial insurance programs, and ongoing work is required to update this practice. Workflows for timing of the collection of patient-reported outcomes are under development. In 2016, the division implemented an online platform at university-based sites called Rheum4U [51] for quality improvement and research that captures patient-reported functional status and composite measures of disease activity. Patients are instructed to fill out their outcomes one week prior to clinic assessment so they are available for rheumatologist review at the point of care. Individuals who are not consented to the platform continue to receive paper-based collection on the day of their clinical encounter.

### Shared decision making

At all times, healthcare professionals and patients participate in shared decision-making regarding treatment and care choices, ensuring patients are at the center of their care. This is especially important where there are benefits and harms that patients need to consider, and/or in the setting of uncertain or equivocal evidence. Documented use of decision aids is encouraged. A paper-based, early RA decision aid is available [11], and work is ongoing to implement its use in our clinics. Updated RA guidelines and accompanying decisions aids for key treatment choices are currently in development by the CRA and are anticipated to be available soon for incorporation into our clinical pathway.

### Ongoing care and follow-up

**Fig. 4:**
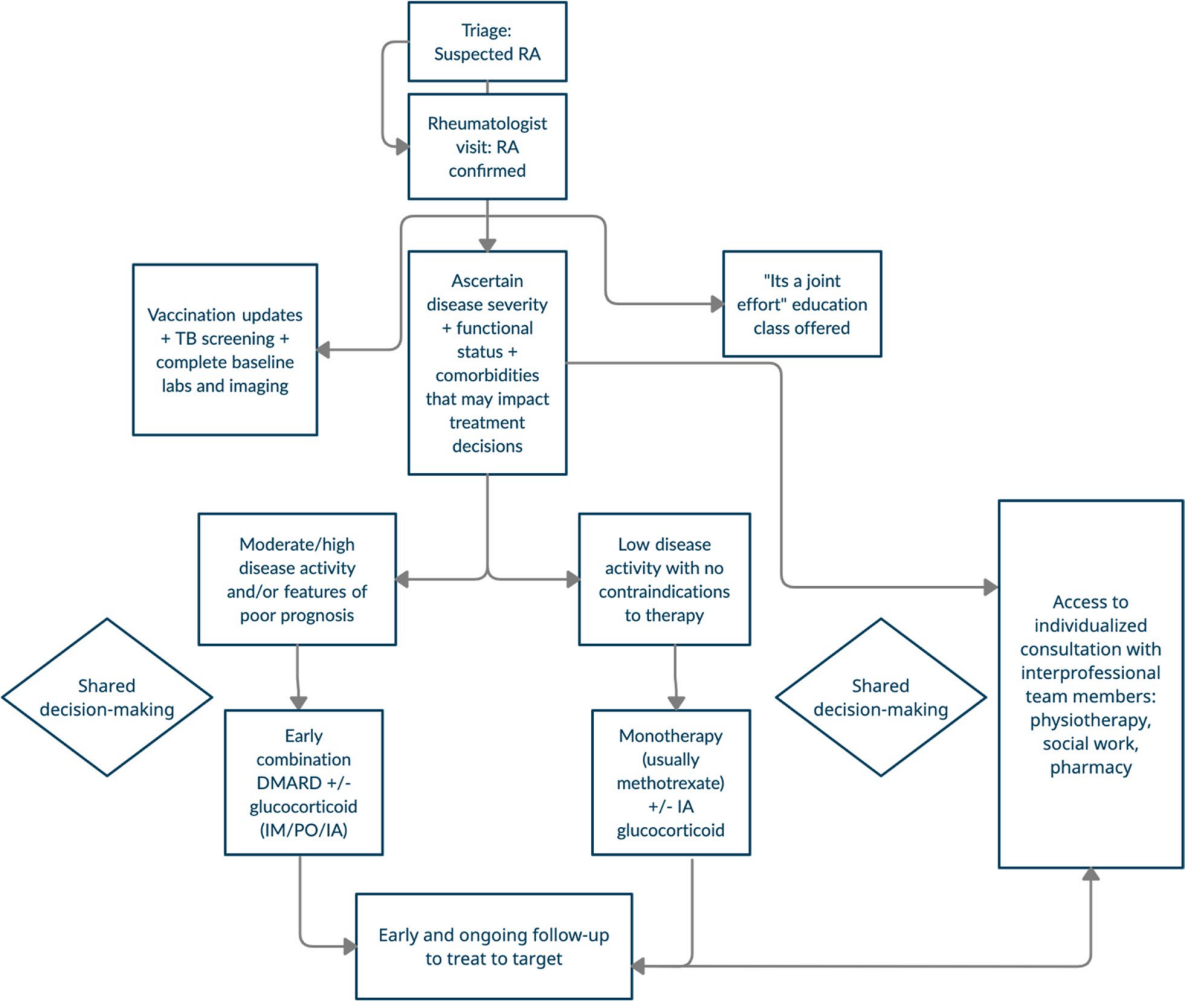
Calgary early rheumatoid arthritis care pathway overview. Intraarticular (IA), intramuscular (IM), oral (po), disease modifying anti-rheumatic drug (DMARD)

Figure 4 provides an overview of the care pathway. Early RA follow-up is within the first 2–4 weeks following diagnosis via telephone (either nurse or physician-led). The purpose of this phone call is to give the patient an opportunity to have their questions and concerns addressed, review any baseline laboratory investigations, and assess tolerability of new medications. For patients with moderate to high disease activity, follow up is recommended every 4–6 weeks to ensure their disease is improving. Once a stable disease is established, patients are seen every 3–6 months for the first year, then every 6 months for year 2, and every 6–12 months thereafter. Laboratory monitoring occurs monthly for the first 3 months (or once a stable medication regimen has been determined), then every 3 months thereafter.

### Nursing protocols

Nursing staff play an integral role in the care of patients with RA. At our centre to facilitate implementation of the care pathway (Phase 4), we have recently implemented a nursing case-based model. In this model of care, a single nurse works closely with 2–3 rheumatologists to help address patients’ needs. All nurses involved have licensed practical nurse/registered nurse (LPN/RN) training and undergo additional structured clinical training on medications, vaccines and other treatments with our clinical pharmacist, physical exam, and musculoskeletal assessment with our physiotherapist, in addition to hands-on training with rheumatologists in clinic. We have created a nursing telephone line and developed a rheumatology clinical guidance document to address common questions. All patients are provided a business card with nursing line information and a general guidance brochure on the clinic resources available to them. The nursing clinical guidance document addresses counselling on strategies for addressing common patient concerns including symptom management including pain management strategies (including non-pharmacologic and over-the-counter medications), and nausea (particularly related to methotrexate and other DMARDs). Nurses are also involved in an educational capacity, teaching patients about post intra-articular injection care, as well as DMARD and biologic education in clinic. They also assist with providing timely advice for when to hold DMARDs during infection and perioperative periods. The divisional rheumatology website is also a resource for patients and healthcare providers.

### Interdisciplinary care

In addition to our nursing staff, our model of care is supported by a clinical pharmacist who is specialized in rheumatology care, a physiotherapist with additional Advanced Clinician Practitioner in Arthritis Care (ACPAC)[52] training and a social worker. In collaboration with rheumatologists and nursing staff, these key team members have developed and run online educational modules for patients (“It’s a Joint Effort”) that cover the basics of diagnosis and treatment of inflammatory arthritis, underline the importance of vaccinations, and educate the patient in coping with their illness, among other topics. Patients who complete the modules can access a virtual live discussion with members of the rheumatology health team for monthly question-and-answer sessions. Individualized care is also available to meet specific patient needs from these healthcare professionals. For example, pharmacist consultation is available to patients who have specific treatment questions, or complex medical needs e.g., patients planning pregnancies or those with comorbidities or hesitancy regarding treatment. Social work consultation is available for individuals needing assistance navigating medication coverage, job and financial impacts of the illness and managing stress and mental health impacts of the disease.

Physiotherapy services are also available to patients, with in-person or virtual physiotherapy available to assist patients in addressing the functional impacts of RA. Specialty rehabilitation services in the community are available for treatment of specific musculoskeletal conditions including rheumatologic diagnoses. The rehabilitation focus is on achieving client goals in improved functionality and independence, to enable return to work and activity. These sessions include one-on-one interventions, education, exercise, self-management strategies, and where appropriate, connection with other community resources. Additional non-rheumatology specific resources are also available to support patients including access to mental health services, dieticians (both group education and individualized referrals) and classes to support exercise and healthy lifestyle choices through regional services.

## Discussion

A care pathway, defined as a statement of goals and key elements of care based on evidence, best practice, and patients’ expectations [53], has the potential to reduce unwarranted variation in care and improve patient outcomes for rheumatologic conditions. Using a practice survey and group discussions with our interdisciplinary team, we drafted a care pathway for the management of early RA, addressing early workup, choice of initial DMARDs, recommended vaccinations, and use of steroids. Our triage system for stratifying the urgency of referrals for early inflammatory arthritis, as well as protocols for our nursing case manager roles, are highlighted in this document along with our interdisciplinary team roles to support optimal patient care. This work represents a formative step to further quality improvement efforts in the division of rheumatology. Implementation of the pathway and developing strategies for evaluation are ongoing to address many of the barriers we have identified through this process including variability of physician practices and resource limitations.

Given the looming shortage of rheumatologists [54] and long waitlists for specialist care, life-long speciality care for all patients with RA may no longer be feasible. Through further integration of RA care within primary care, it may be possible to reduce the need for continuous rheumatology follow-up for patients with stable disease, thereby improving overall system access to early diagnosis and treatment. Having clearly defined care pathways and rapid access to rheumatology specialists for advice are necessary prerequisites to ensure ongoing high-quality care for all patients. Our early RA care pathway represents the first step to a longitudinal care pathway to optimize patient and system health outcomes in RA. While the current care pathway represents primarily rheumatology-specific practices at present, in future, we aim to develop strategies for improved shared-care with primary care practice. For example, individuals with stable RA in long-term remission on conventional synthetic DMARDs or in drug-free remission could be returned to primary care with clear guidance for ongoing management and means of accessing specialty care when required. Such pathways may improve access to timely care and treatment provincially; however, such strategies need to be monitored to ensure quality of care is maintained for all individuals with RA.

While clinical guidelines often focus on treatment recommendations, an important aspect of the proposed pathway is the emphasis on interdisciplinary care. Teams consisting of nurses, physiotherapists, occupational therapists, dietitians, and other allied health professionals, can address a range of patient concerns, including those related to medication side effects, questions regarding abnormal lab results, psychological support, and self-management of arthritis flares. This builds on evidence that involving interdisciplinary health professionals in patient education and self-management can decrease healthcare costs [55] and lead to dramatic improvements in patient satisfaction with care [55, 56]. The importance of patient involvement in health care was also highlighted in the 2021 EULAR recommendations for the implementation of self-management strategies in patients with inflammatory arthritis [57]. It focuses on patient education and self-management interventions such as problem solving, goal setting and cognitive behavioural therapy, recognizing the role of patient organizations and healthcare providers in helping patients access these resources. Unfortunately, access to interdisciplinary care in RA remains fragmented and often underfunded. A robust care team requires resources for hiring, interprofessional education, and supervision of skilled allied staff, which may not be feasible at every care centre.

A key aspect of implementation of a care pathway is measurement and evaluation of system and health outcomes [58]. Data gathered locally can be used to obtain key measures such as time to diagnosis and rates of patient participation in care [30], as well as frequency of follow-up and documentation of disease activity [31]. Our clinic has implemented a starter set of measures capturing treat-to-target concepts for continuous measurement (Additional file 1: Table S9) [31], and implementation of a more comprehensive measurement framework is planned to evaluate care pathway implementation. Data gathering and creation of individualized physician reports based on local data was perceived as valuable by clinicians [31], and helped contribute to long-term quality improvement. Additionally, we have developed key performance indicators for centralized intake systems for arthritis care [27], and ongoing evaluation of access to care is planned following implementation of a new EMR in our healthcare system.

While the proposed care pathway represents a concerted local effort to improve the quality of early RA care, there are important limitations to discuss. Firstly, the care pathway describes our local practice, and all aspects may not be readily or easily adopted in all settings depending on resource constraints. The pathway also does not completely address virtual care as this is an evolving area in rheumatology. The CRA has recently developed some Best Practice Statements [59] to support virtual care in rheumatology that we are incorporating in the pathway. Our pathway development did not involve a formal consensus process such as a modified Delphi as this is typically run over multiple rounds, can be time consuming, and difficult to execute, given the busy clinical practices of all individuals involved. Instead, we ensured a working group with diverse perspectives and clinical roles was involved in drafting the document, including the division chief, and nursing leads key to implementation. As such, it is possible that some physicians may disagree or not be completely adherent with some of the principles outlined in this document. Also, current monitoring of implementation of this pathway is limited due to challenges with data acquisition, which we anticipate will improve with a new provincial EMR. Lastly, while there was no process to implement patient feedback into the current document; this will be considered for future iterations.

## Conclusions

In summary, the proposed care pathway highlights an approach to patient-centered early RA care using an interdisciplinary approach. Our care pathway will be updated over time based on emerging guidelines, best practices, and local data. We also plan to expand it to incorporate care strategies for patients with a stable disease course to better allow primary care physicians to resume care when appropriate to improve capacity. Implementation of our pathway is ongoing, as are strategies for continuous evaluation.

## Supplementary Information

**Additional file 1.** Practice pattern survey results (Tables 1–7), facilitators and barriers of care pathway component by Consolidated Framework for Implementation Research (CFRI) domain (Table 8), and currently collected performance measures (Table 9).

## Data Availability

The care pathway and associated documents are available upon reasonable request from the senior author (Dr Claire Barber, cehbarbe@ucalgary.ca).

## Abbreviations

ACR: American college of rheumatology
Anti-CCP: Anti-cyclic citrullinated peptide antibody
ACPAC: Advanced clinician practitioner in arthritis care
CBC: Complete blood count
CRP: C-reactive protein
CRA: Canadian Rheumatology Association
CLINHAQ: Clinical health assessment questionnaire
DMARD: Disease modifying anti-rheumatic drug
DAS28: Disease activity score 28
ESR: Erythrocyte sedimentation rate
EULAR: European League Against Rheumatism
HAQ-DI: Health assessment questionnaire disability index
HAQ-II: Health assessment questionnaire II
LPN: Licensed practical nurse
PROMIS PF10a: PROMIS physical function 10 item short form
RN: Registered nurse
RA: Rheumatoid arthritis
RF: Rheumatoid factor
TB: Tuberculosis
