# Supplementary material for: Development of an interdisciplinary early rheumatoid arthritis care pathway
Source: BMC Rheumatol. 2022 Jun 25;6:35. doi: 10.1186/s41927-022-00267-x (PMC9233314; doi:10.1186/s41927-022-00267-x)
Supplement: Supplementary file 1 — Additional file 1. Practice pattern survey results (Tables 1–7), facilitators and barriers of care pathway component by Consolidated Framework for Implementation Research (CFRI) domain (Table 8), and currently collected performance measures (Table 9). [file 41927_2022_267_MOESM1_ESM.docx]

Supplemental material

Rheumatologist Practice Pattern Survey Results

| Supplemental Table 1. Rheumatologist self-reported likelihood of prescribing DMARDs to patients with a new diagnosis of rheumatoid arthritis | | | | | | | | |
| --- | --- | --- | --- | --- | --- | --- | --- | --- |
|  | Low disease activity | | |  | Moderate to high disease activity | | | |
| Treatment | n | Median  (Q1, Q3) | Range  (min, max) |  | n | Median  (Q1, Q3) | Range  (min, max) |  |
| DMARD monotherapies |  |  |  |  |  |  |  |  |
| MTX | 15 | 6.0 (6.00, 7.25) | 1, 9 |  | 15 | 4.5 (1.00, 5.25) | 1, 9 |  |
| HCQ | 15 | 4.5 (3.00, 8.25) | 1, 9 |  | 15 | 1.0 (1.00, 1.25) | 1, 3 |  |
| SSZ | 15 | 1.5 (1.00, 3.25) | 1, 5 |  | 15 | 1.0 (1.00, 1.00) | 1, 3 |  |
| LFD | 14 | 1.0 (1.00, 1.00) | 1, 5 |  | 15 | 1.0 (1.00, 1.25) | 1, 4 |  |
| DMARD combination therapies |  |  |  |  |  |  |  |  |
| MTX + HCQ | 15 | 6.5 (2.75, 9.00) | 1, 9 |  | 15 | 9.0 (7.75, 9.00) | 1, 9 |  |
| MTX + SSZ | 14 | 1.0 (1.00, 4.00) | 1, 6 |  | 15 | 1.0 (1.00, 3.25) | 1, 7 |  |
| MTX + LFD | 14 | 1.0 (1.00, 1.00) | 1, 4 |  | 15 | 1.0 (1.00, 2.50) | 1, 6 |  |
| HCQ + SSZ | 14 | 1.0 (1.00, 2.50) | 1, 4 |  | 15 | 1.0 (1.00, 2.00) | 1, 4 |  |
| HCQ + LFD | 14 | 1.0 (1.00, 1.00) | 1, 4 |  | 12 | 1.0 (1.00, 1.00) | 1, 4 |  |
| MTX + HCQ + SSZ | 14 | 1.0 (1.00, 1.00) | 1, 6 |  | 13 | 7.0 (2.50, 8.00) | 1, 9 |  |
| MTX + HCQ + LFD | 14 | 1.0 (1.00, 1.00) | 1, 2 |  | 13 | 1.0 (1.00, 1.50) | 1, 7 |  |
| MTX + SSZ + LFD | 14 | 1.0 (1.00, 1.00) | 1, 2 |  | 13 | 1.0 (1.00, 1.00) | 1, 7 |  |
| Responses were provided on a scale from 1 = Not very likely to 9 = Very likely. HCQ = hydroxychloroquine, LFD = leflunomide, MTX = methotrexate, SSZ = sulfasalazine. | | | | | | | | |

| Supplemental Table 2. Rheumatologist self-reported likelihood of prescribing different initial disease-modifying antirheumatic drug treatments for patients with a new diagnosis of rheumatoid arthritis | | | |
| --- | --- | --- | --- |
| Treatment | N | Median (Q1, Q3) | Range (min, max) |
| Methotrexate |  |  |  |
| Oral | 16 | 5.5 (5.00, 7.00) | 2, 9 |
| 10mg | 14 | 1.0 (1.00, 1.00) | 1, 4 |
| 15mg | 13 | 5.0 (3.00, 7.00) | 1, 9 |
| 20mg | 15 | 9.0 (8.00, 9.00) | 1, 9 |
| 25mg | 15 | 7.0 (6.00, 8.00) | 1, 9 |
| Subcutaneous, pre-filled syringe | 16 | 7.5 (5.25, 9.00) | 2, 9 |
| Subcutaneous, vial | 16 | 7.0 (5.00, 8.00) | 1, 9 |
| 10mg | 13 | 1.0 (1.00, 1.00) | 1, 3 |
| 15mg | 12 | 4.0 (2.75, 5.00) | 1, 7 |
| 20mg | 14 | 9.0 (9.00, 9.00) | 7, 9 |
| 25mg | 15 | 8.0 (7.00, 8.00) | 2, 9 |
| Sulfasalazine |  |  |  |
| Initial titration |  |  |  |
| 500mg BID increased to 1000mg BID after 2 weeks | 15 | 1.0 (1.00, 4.00) | 1, 9 |
| 500mg OD increased by 500mg weekly until target dose is reached | 15 | 9.0 (2.50, 9.00) | 1, 9 |
| Other | 10 | 1.0 (1.00, 1.00) | 1, 9 |
| Target dose |  |  |  |
| 500mg daily | 14 | 1.0 (1.00, 1.75) | 1, 9 |
| 1000mg daily | 13 | 3.0 (1.00, 5.00) | 1, 8 |
| 1500mg daily | 13 | 2.0 (1.00, 4.00) | 1, 8 |
| 2000mg daily | 14 | 9.0 (7.50, 9.00) | 1, 9 |
| More than 200mg daily | 14 | 9.0 (7.50, 9.00) | 1, 9 |
| Hydroxychloroquine |  |  |  |
| 5mg/kg | 15 | 9.0 (1.50, 9.00) | 1, 9 |
| 400mg daily then decreased to 5mg/kg | 15 | 7.0 (1.00, 9.00) | 1, 9 |
| Other | 3 | 1.0 (1.00, 1.00) | 1, 1 |
| Responses were provided on a scale from 1 = Not very likely to 9 = Very likely. Subcutaneous methotrexate dosages are for either pre-filled syringe or vial. BID = bidaily, OD = once daily. | | | |

| Supplemental Table 3. Rheumatologist self-reported likelihood of prescribing different routes of corticosteroid administration to patients with a new diagnosis of rheumatoid arthritis | | | | | | |
| --- | --- | --- | --- | --- | --- | --- |
|  | Low disease activity | |  | Moderate to high disease activity | | |
| Treatment | Median (Q1, Q3) | Range (min, max) |  | Median (Q1, Q3) | Range (min, max) |  |
| Oral | 1.0 (1.00, 1.50) | 1, 5 |  | 5.0 (3.50, 5.00) | 1, 8 |  |
| Intramuscular | 4.0 (2.00, 6.00) | 1, 9 |  | 9.0 (8.00, 9.00) | 1, 9 |  |
| Intra-articular | 5.0 (3.00, 7.00) | 1, 9 |  | 6.0 (3.00, 8.50) | 1, 9 |  |
| Responses were provided on a scale from 1 = Not very likely to 9 = Very likely | | | | | | |

| Supplemental Table 4. Rheumatologist self-reported likelihood of follow up appointment timing for patients with a new diagnosis of rheumatoid arthritis | | | | | | | | |
| --- | --- | --- | --- | --- | --- | --- | --- | --- |
|  | Low disease activity | | |  | Moderate to high disease activity | | | |
| Follow up | n | Median  (Q1, Q3) | Range  (min, max) |  | n | Median  (Q1, Q3) | Range  (min, max) |  |
| Less than 6 weeks | 13 | 1.0 (1.00, 2.00) | 1, 5 |  | 14 | 3.5 (1.25, 5.75) | 1, 9 |  |
| 6 weeks | 13 | 4.0 (1.00, 5.00) | 1, 9 |  | 15 | 7.0 (4.50, 9.00) | 1, 9 |  |
| 3 months | 15 | 9.0 (8.00, 9.00) | 7, 9 |  | 15 | 8.0 (5.00, 9.00) | 1, 9 |  |
| 4 months | 14 | 4.5 (1.00, 7.00) | 1, 9 |  | 14 | 2.0 (1.00, 2.00) | 1, 5 |  |
| Greater than 4 months | 13 | 1.0 (1.00, 2.00) | 1, 5 |  | 14 | 1.0 (1.00, 1.00) | 1, 3 |  |
| Responses were provided on a scale from 1 = Not very likely to 9 = Very likely | | | | | | | | |

| Supplemental Table 5. Descriptive characteristics of rheumatologist prescription decisions for patients with a new diagnosis of rheumatoid arthritis | | |
| --- | --- | --- |
| Characteristics | N | n (%) |
| Factors considered when selecting methotrexate route |  |  |
| Patient preference | 16 | 16 (100) |
| Efficacy | 16 | 12 (75) |
| Complexity of drug regime | 16 | 7 (44) |
| Cost | 16 | 7 (44) |
| Other | 16 | 4 (25) |
| Use of titration when starting methotrexate (yes/no) |  |  |
| Oral | 16 | 8 (50) |
| Subcutaneous | 16 | 5 (31) |
| Methotrexate target dosing |  |  |
| Varies based on patient factors | 15 | 11 (73) |
| Factors considered when varying dose |  |  |
| Renal function | 11 | 11 (100) |
| Age | 11 | 7 (64) |
| Weight | 11 | 4 (36) |
| Ethnicity | 11 | 4 (36) |
| Other comorbidities | 11 | 6 (55) |
| Other factors | 11 | 4 (36) |
| Folic acid dosing |  |  |
| 1mg weekly | 15 | 7 (47) |
| 5mg daily | 15 | 6 (40) |
| 5mg 5/7 days per week | 15 | 2 (13) |
| Typical intramuscular corticosteroid dosing |  |  |
| 60mg | 15 | 1 (7) |
| 80mg | 15 | 7 (47) |
| 120mg | 15 | 3 (20) |
| Other | 15 | 4 (27) |
| Factors considered when varying dose of corticosteroid |  |  |
| Disease activity | 15 | 13 (87) |
| Weight | 15 | 12 (80) |
| Diabetes | 15 | 11 (73) |
| Mental health | 15 | 10 (67) |
| Osteoporosis | 15 | 7 (47) |
| Age | 15 | 6 (40) |
| Other comorbidities | 15 | 8 (53) |
| Other factors | 15 | 1 (7) |
| None | 15 | 1 (7) |

| Supplemental Table 6. Self-reported rheumatologist practices for laboratory monitoring of patients with a new diagnosis of rheumatoid arthritis | | | |
| --- | --- | --- | --- |
| Laboratory Test | Baseline, n (%) |  | Monitoring, n (%) |
| Bloodwork |  |  |  |
| CBC | 14 (93) |  | 15 (100) |
| Albumin | 13 (87) |  | 13 (87) |
| ALP | 10 (67) |  | 10 (67) |
| ALT | 15 (100) |  | 15 (100) |
| AST | 5 (33) |  | 4 (27) |
| GGT | 2 (13) |  | 0 (0) |
| Creatinine | 15 (100) |  | 15 (100) |
| ANA | 12 (80) |  | - |
| Anti-CCP | 15 (100) |  | - |
| Anti-dsDNA | 1 (7) |  | - |
| ENA | 6 (40) |  | - |
| Rheumatoid factor | 14 (93) |  | - |
| CRP | 15 (100) |  | 15 (100) |
| ESR | 6 (40) |  | 4 (27) |
| Electrolytes | 0 (0) |  | 0 (0) |
| HCV | 13 (87) |  | - |
| Hep B core Ab | 13 (87) |  | - |
| Hep B sAb | 13 (87) |  | - |
| Hep B sAg | 15 (100) |  | - |
| HIV | 5 (33) |  | - |
| Immunoglobulins | 4 (27) |  | - |
| SPEP | 2 (13) |  | - |
| Urate | 6 (40) |  | - |
| Urinalysis | 6 (40) |  | - |
| Other | 1 (7) |  | 0 (0) |
| X-Rays |  |  |  |
| Chest | 14 (93) |  | - |
| Hands/wrists | 14 (93) |  | - |
| Feet/ankles | 13 (87) |  | - |
| C-Spine | 0 (0) |  | - |
| Other | 2 (13) |  | - |
| – item was not presented to participants as a response option for the survey question. n = 15.  alkaline phosphatase (ALP), alanine transaminase (ALT), aspartate aminotransferase (AST), anti-nuclear antibody (ANA), anti-cyclic citrullinated peptide antibody (anti-CCP), anti-double stranded DNA (anti-dsDNA), cervical spine (C-spine), c-reactive protein (CRP), erythrocyte sedimentation rate (ESR), extractable nuclear antigens (ENA), Hepatitis C Virus (HCV), Hepatitis B core antibodies (Hep B Core Ab), Hepatitis B surface antibodies (Hep B sAb), Hepatitis B surface antigen (Hep B sAg), Human Immunodeficiency Virus (HIV), serum protein electrophoresis (SPEP) | | | |

| Supplemental Table 7. Self-reported rheumatologist practices for drug specific laboratory monitoring, vaccinations, and medication counseling for patients with a new diagnosis of rheumatoid arthritis | |
| --- | --- |
| Characteristics | n (%) |
| Frequency of bloodwork for monitoring methotrexate |  |
| Initially |  |
| Monthly | 15 (100) |
| Once stable |  |
| Every 2 months | 1 (7) |
| Every 3 months | 14 (93) |
| Different frequency of bloodwork for drugs other than methotrexate |  |
| Yes | 7 (47) |
| Timing of TB screening |  |
| Time of RA diagnosis | 6 (40) |
| Prior to initiation is biologics | 8 (53) |
| Other | 1 (7) |
| Approach to vaccines |  |
| Refer to public health clinic | 10 (67) |
| Provide specific recommendations to patient | 6 (40) |
| Refer to their general practitioner | 5 (33) |
| Other | 3 (20) |
| Who typically provides medication counseling |  |
| Myself | 15 (100) |
| RPN | 4 (27) |
| Pharmacist | 4 (27) |
| LPN | 3 (20) |
| Other | 1 (7) |
| Routinely used medication counseling resources |  |
| *RheumInfo* medication handouts | 14 (93) |
| *It’s a Joint Effort* class | 10 (67) |
| *Methotrexate Injection* class | 9 (60) |
| Other | 1 (7) |
| n = 15. LPN = licensed practical nurse, RPN = registered practical nurse, RA = rheumatoid arthritis, TB = tuberculosis. | |

Supplemental Table 8. Facilitators and Barriers of Care Pathway Component by Consolidated Framework for Implementation Research (CFRI) Domain

| **CFIR domain** | **Main care pathway component** | | | | | |
| --- | --- | --- | --- | --- | --- | --- |
|  | Triage process | Early RA investigations | Vaccinations | Early RA treatment | Shared Decision-Making | Interdisciplinary care |
| **Innovation characteristics** | | | | | | |
| ***Facilitators*** | | | | | | |
| Strong evidence base | + |  | + | + |  |  |
| Perception of advantage of innovation | + |  | + |  |  |  |
| Ability to test innovation on small scale (trialability) |  | + | + | + | + | + |
| ***Barriers*** | | | | | | |
| Complexity of pathway component | + |  | + | + | + | + |
| Variability regarding evidence base/standard of care |  | + |  |  |  |  |
| Cost (often in adequate staffing) | + |  | + |  | + | + |
| **External environment and context (Outer setting)** | | | | | |  |
| ***Facilitators*** | | | | | |  |
| Regional experience with care pathway component | + |  | + |  |  | + |
| Access to provincial health record | + | + | + |  |  |  |
| Addresses complex patient needs | + | + | + | + | + | + |
| Improves shared care with primary care provider | + | + | + |  |  |  |
| ***Barriers*** | | | | | |  |
| Lack of standardization of care pathway component across province | + | + |  | + | + | + |
| Unable to embed care pathway component into current EMR systems |  | + | + | + | + |  |
| **Internal context and setting** | | | | | | |
| ***Facilitators*** | | | | | | |
| Compatible with current workflows/systems | + | + | + | + |  | + |
| Divisional commitment to providing high quality care | + | + | + | + |  | + |
| Some outcome measure collection and quality reporting through local quality improvement and research databases (Rheum4U) |  |  |  | + |  |  |
| ***Barriers*** | | | | | | |
| Variability of resources (e.g., nursing support) in community vs university-based clinics and between university-based clinics | + | + | + | + | + | + |
| Different EMR in university-based vs community clinics | + | + |  | + | + |  |
| Challenges in entering and accessing data to measuring and reporting back on quality of care/pathway component | + | + | + | + | + | + |
| **Characteristics of individuals** | | | | | | |
| ***Facilitators*** | | | | | | |
| Common interest and desire to provide high quality care | + | + | + | + | + | + |
| ***Barriers*** | | | | |  |  |
| Existing practice variation between physicians |  | + | + | + | + | + |
| Clinician skepticism regarding a need for change |  | + |  |  | + |  |
| Different staff/managerial structures at different sites | + |  |  |  |  | + |
| **Process** | | | | | | |
| ***Facilitators*** | | | | | | |
| Future implementation of provincial EMR with potential for shared provincial pathway and tools, and tracking implementation | + | + | + | + | + | + |
| Local champions with expertise in quality measurement, improvement, and guideline development | + | + | + | + | + | + |
| Access to provincial and local expertise in implementation | + | + | + | + | + | + |
| Involvement of key stakeholders (nursing staff/managers) to facilitate implementation | + | + | + | + | + | + |
| ***Barriers*** | | | | | | |
| The new provincial EMR will not be implemented in most community practices | + | + |  | + | + |  |
| Implementation likely largely to be at university-based sites given resourcing and tracking ability | + | + |  | + | + |  |
| No formal quality review structure at a divisional level | + | + | + | + | + | + |
| Lack of patient advisory council | + | + | + | + | + | + |
| Electronic medical record (EMR); Shared Decision Making (SDM). For each pathway component, + is used to denote identified facilitators or barriers | | | | | | |

| Supplemental Table 9. Currently collected treat-to-target performance measures |
| --- |
| **Performance measure^*^** |
| Percentage of RA patients seen in follow-up at least once per year |
| Percentage of patients with $\geq$50% of total number of outpatient encounters per year with assessment of disease activity using a standardized measure^**^ |
| Percentage of RA patients seen in clinic within 3 months if not in remission |
| Percentage of RA patients with active RA who have low disease activity within 6 months |
| Percentage of RA patients in remission during the year |
| Rheumatoid arthritis (RA).  **Further operationalization and early reporting of these performance measures is reported in Rheumatol Ther (2020) 7:909–925 <https://doi.org/10.1007/s40744-020-00237-0>. Initial development of the measures is described J Rheumatol 2016 Vol. 43 Issue 3 Pages 530-40 DOI: 10.3899/jrheum.150839  *Standardized measures include Disease activity score 28 erythrocyte sedimentation rate (DAS28 ESR) or DAS28 C-reactive protein (DAS28 CRP) or clinical disease activity index (CDAI) |
